# Mediating Effect of Social Support in the Relationship Between Childhood Abuse and Non-Suicidal Self-Injury Among Chinese Undergraduates: The Role of Only-Child Status

**DOI:** 10.3390/ijerph16204023

**Published:** 2019-10-21

**Authors:** Huiqiong Xu, Xianbing Song, Shanshan Wang, Shichen Zhang, Shaojun Xu, Yuhui Wan

**Affiliations:** 1 Department of Maternal, Child & Adolescent Health, School of Public Health, Anhui Medical University, 81 Meishan Road, Hefei 230032, Anhui, China; xhqxuhuiqiong@163.com (H.X.); 13625510589@163.com (S.W.); zhangshichen@ahmu.edu.cn (S.Z.); 2 Department of Basic Course, Anhui medical College, 632 Furong Road, Hefei 230601, Anhui, China; sxbayd@163.com; 3 Anhui Provincial Key Laboratory of Population Health &Aristogenics, 81 Meishan Road, Hefei 230032, Anhui, China

**Keywords:** child abuse, social support, non-suicidal self-injury, college students

## Abstract

Previous research has found a relationship between child abuse and non-suicidal self-injury (NSSI). However, few studies have examined the role of social support underlying this association. Moreover, the influence of the only child status on the mediating effect of social support has not been studied yet. The aim of this study was to investigate the mediating role of social support on the association between specific forms of child abuse and NSSI as well as the role of the only child status on the mediated pathways, among undergraduates. A total of 4799 participants were selected from two medical colleges in the Anhui province using stratified cluster sampling. Pearson's correlation analysis was used in analyzing the relationship. Bootstrapping procedures were applied to examine the mediating effects. After adjusting for confounders, the results showed that the mediating effect of social support on the association between childhood abuse and NSSI was not significant in the total sample. However, among only children, the mediating effects of social support between overall childhood abuse, physical abuse, emotional abuse, and NSSI were 9.65%, 14.82%, and 8.12%, respectively. Moreover, the mediating effect of social support from family and relatives was relatively higher than that from other sources. Social support had a mediating effect on the relationship between childhood abuse and NSSI among only-children. The enhancing of social support may contribute to the prevention and control of NSSI for those who were only-children in undergraduates, especially those who have experienced childhood abuse.

## 1. Introduction

Non-suicidal self-injury (NSSI) is interpreted as the deliberate, self-inflicted damage of body tissue without suicidal intent and for purposes not socially or culturally sanctioned [1]. It is a major public health problem in adolescents and young adults worldwide; approximately 17% to 38% of adolescent samples report that they have engaged in at least one incident of NSSI at some point in their lives [2,3,4], with even greater rates reported in clinical populations [5,6]. Several studies have found that a prior history of NSSI has been identified as one of the strongest predictors of suicidal thoughts and behaviors [7,8].

Furthermore, child abuse has often been identified as a risk factor for NSSI [9,10]. A systematic review of 26 studies about the relationship between childhood maltreatment and NSSI indicated that the increased vulnerability to NSSI seems to be related to experiences of childhood maltreatment, particularly sexual abuse [11]. The data from the Minnesota Longitudinal Study of Risk and Adaptation prospectively documented that childhood experiences of abuse/neglect were associated with more frequent and severe NSSI up 26 years of age [12]. It is also showed that students with higher levels of childhood adversity felt more stressed and less supported than those with lower levels [13]. As Herrenkohl’s research expected, abused children appear more susceptible to experience poorer relationships and less social support, which can promote health problems and worsen conditions among those who are ill [14]. One settings study in community and in-patient mental health also showed that children and adolescents who had some form of social support had a 26% decrease in the odds of engaging in NSSI when compared with their counterparts who lacked social support [15].

From what has been discussed above, we consider that social support might mediate the association between childhood abuse and NSSI. Some scholars have shown that social support is an important mental health resource so far, which plays a buffering effect on the relationship between childhood abuse and adverse outcomes [16,17]. Christoffersen et al. [18] tested whether social support could act as a mediator between NSSI and previous experiences with childhood maltreatment in adolescence. And Evans et al. [19] also suggested that as the severity of maltreatment (physical and emotional abuse, emotional neglect) increased, the buffering effect of perceived social support from family on trauma symptoms diminished. However, none of the above studies specifically analyzed the mediating role of social support between different childhood abuse subtypes and NSSI. Moreover, college is a transitional period and full of stress, which may be associated with adverse childhood experiences and exacerbate their effects. College students, as a relatively independent group who are more capable of self-management, have a stronger sense of autonomy and independent living habits than middle school students [20]. It is not clear whether social support plays a mediating role between childhood abuse and NSSI in college students.

Research has shown that those college students who are only-children are more likely to be spoiled at home, and spoiled children have higher emotional expectations from society [21]. With the same emotional support, the subjective feelings of only children are less satisfied than those of non-only children. At the same time, the lack of brothers and sisters makes them lose the natural support of their peers. In China, only-children often miss out on important opportunities to rehearse some of the more complicated aspects of relationships within a safe environment, as well as opportunities to develop psychosocial skills, emotional support, and learning opportunities that can be provided by siblings [22]. Thus, in the face of childhood abuse, only-children may be more vulnerable and more readily engage in NSSI. Finally, despite evidence differentiating only-children and non-only children in terms of the severity of child abuse, the feelings or needs of social support and the prevalence of NSSI [20,21,22], few studies have been undertaken to examine the only-child status differences on the mediating effect of social support between child abuse and NSSI, which needs to be a further clarified. This is particularly important in China, since the implementation of the special only-child policy 30 years ago. According to the data from the sixth census in 2010, the number of only children reached 164 million, and this number is on the rise [23]. Particularly, there are many only children in the younger generation, where there is also a dearth of related research. The cultural context of China can help to give a deeper understanding of NSSI that we do not see in other countries.

The present study first sought to explore whether social support mediates the relationship between different types of childhood abuse and NSSI among college students in China, and then examine whether the roles varied by the only child status. We hypothesize that increased social support could reduce the risk of developing NSSI in children who experience various types of childhood abuse. Further, we explored whether there are differences among those who were only-children or not.

## 2. Methods

### 2.1. Participants

Respondents were interviewed for data collection between September and October 2017. A stratified cluster sampling method was used to select 5085 students, involving freshmen and sophomores, recruited from two medical colleges in the Anhui province. According to the professional distribution of each grade, 172 classes were randomly selected; all the students in the classes were included in this health survey and were asked to complete an anonymous questionnaire. The research content and data collection procedures were approved by the Ethics Committee of Anhui Medical University (20170290). Of the 5085 sampled students, 286 were excluded from the study because they were either unwilling to complete the questionnaire, had high levels of missing data, or had obvious logical errors or inconsistent responses. For example, participants answered that they had harmed themselves in the past 12 months, but also filled the frequency of NSSI was 0. In this case, the sample was deleted. The effective response rate was 94.4%. In total, 4799 students, mean aged 20.51 years (SD = 1.02), were included in the study, and 1577 participants (32.9%) were an only child.

### 2.2. Measures

#### 2.2.1. Non-Suicidal Self-Injury

All participants received a screening questionnaire for NSSI, asking: “In the past 12 months, have you ever intentionally hurt yourself, but not for the purpose of suicide?” A list of several NSSI methods were specified: hit yourself with your fists or palms, pulled your own hair, banged your head or fist against something hard, pinched or scratched yourself, bitten yourself, cut or pierced yourself [24,25]. For those who admitted that they had engaged in NSSI, the frequency of NSSI was asked. The number of occurrences of each NSSI was calculated as the total frequency of NSSI. The internal consistency reliability of NSSI was 0.780 in the current study.

#### 2.2.2. Childhood Abuse

Childhood abuse, including physical abuse, emotional abuse, and sexual abuse, was assessed using the Childhood Abuse Questionnaire [26]. All of the questions used to represent abusive childhood experiences were introduced with the phrase “While you were growing up (during your first 18 years of life), how often did someone do any of these things to you—very often, often, sometimes, occasionally, or never?” Analyses were conducted with 5 categories of summed scores; the higher the total score, the worse the abuse. In the present study, Cronbach’s α coefficient for the physical, emotional, and sexual abuse subscales and the overall scale were 0.806, 0.746, 0.723, and 0.871, respectively.

#### 2.2.3. Social Support

Social support was assessed using the 17-item Social Support Scale for University Students [27], which evaluated three aspects in the past year: classmates and friends’ support, family and relatives’ support, and others’ support. Participants indicated agreement with statements related to social support (e.g., “when I am in trouble, I often turn to my family and relatives for help”) using 5-point Likert responses (“inconformity”, “little inconformity”, “uncertainty”, “little conformity”, “conformity”). The scores for all items were then added to derive an overall social support score that ranged from 17 to 85, had good internal consistency in the current study, with a significant Cronbach’s α coefficient of 0.946. The scale was interpreted as: the higher the score, the better the social support status.

#### 2.2.4. Covariates

Demographic indicators for each participant was noted, including age, gender (male or female), urban/rural residency, only child status, parents’ education level (less than junior middle school, junior middle school, senior middle school, college or higher) and self-perceived economic status of the family (poor, moderate, or good). Psychological symptoms, including emotional, behavioral, and social adaptation symptoms, were evaluated using the psychological domain of the Multidimensional Sub-Health Questionnaire of Adolescents (MSQA) [28]. Cronbach’s α of this scale was 0.809 in the present study.

### 2.3. Data Analysis

All data were entered through Epidata 3.1 and analyzed in SPSS 23.0. First, we did preliminary analyses to examine the differences in partial variables by only child status. Next, we conducted Pearson correlations to test the associations among different types of childhood abuse, social support, and NSSI frequency. To test the potential mediating roles of the social support in the relationship between childhood abuse and NSSI, we conducted a series of mediation analyses following the recommendation of Preacher and Hayes, using bootstrapping procedures to compute 95% bias-corrected confidence intervals around the indirect effect [29]. The indirect effect was tested using a bootstrap estimation approach with 5000 samples; the confidence intervals (CI) that did not contain zero are considered to reflect significant mediation. Finally, we explored whether social support mediated the relationship between the different types of childhood abuse and NSSI, and the extent to which this relationship was moderated by only-child status among college students. The proportion mediated was calculated for all significant associations; this method directly tests the significance of the mediated pathway utilizing results of linear regression analyses.

**Figure 1:**
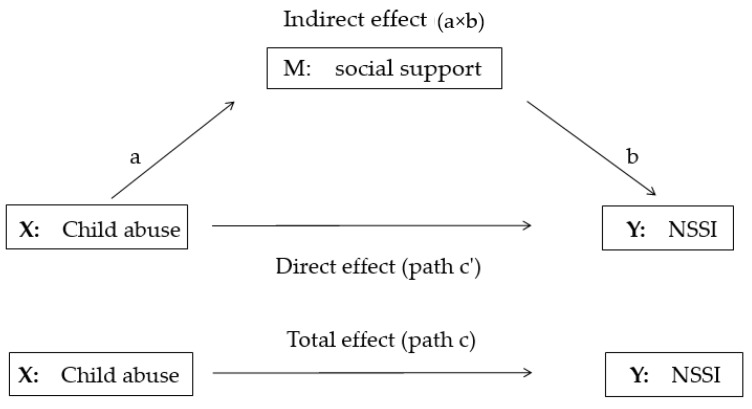
The mediation model.

The mediation model, as shown in Figure 1, presents the three regression equations in the present study. The first regression equation (path c) shows the total effect of the independent variable (X: Child abuse) on the dependent variable (Y: NSSI) through M; and the second equation (path a) shows the association between independent variable (X) and the mediator (M: Social support). The third equation regresses the dependent variable on the independent variable (path c’) and the mediator (path b). Path c’ shows the direct effect of the independent variable (X) on the dependent variable (Y), path b shows the association between the mediator (M) and the dependent variable (Y), a × b indicates the indirect effect of the independent variable (X) on the dependent variable (Y) through the mediator (M).

## 3. Results

### 3.1. Characteristics of Participants

A total of 568 (11.8%) students reported that they had engaged in NSSI during the previous 12 months. The rate of NSSI in only children was higher than that of non-only children (13.4% vs 11.1%). Compared to non-only children, only-children had a lower level of social support, and they were more prone to NSSI, and the difference was statistically significant (*p* < 0.05). The results show that only-children are more likely to be boys, in an urban area, have higher educated parents, and live in higher-income families (*p* < 0.001). The details of socio-demographic factors by only-child status can be seen in Table 1.

### 3.2. Correlation Analysis of Child Abuse Score, Social Support Score, and NSSI Frequency

A correlation matrix of the study variables is presented in Table 2. In the total sample, childhood abuse, physical, emotional, and sexual abuse scores were positively correlated with NSSI frequency (*p* < 0.01) and negatively correlated with social support scores (*p* < 0.01). The social support scores were negatively correlated with NSSI (*r* = −0.11, *p* < 0.01) in the only-child status, with one notable exception for failing to support a link between sexual abuse scores and social support scores.

### 3.3. Testing for Mediation Effects of Social Support in the Relationship Between Childhood Abuse and NSSI

The Bootstrap method was used to test the direct and indirect effects. The results are shown in Table 3, Table 4 and Table 5. In the total sample, the mediating effect of social support on the association between childhood abuse and NSSI was 3.91% (*p* < 0.001) in the undergraduate sample. Further, the effects of physical, emotional, and sexual abuse were 5.46%, 3.62%, and 6.97% (*p* < 0.001), respectively. In adjusted models, the mediating effect of social support was not significant. Among only children, social support reduced the direct effect of childhood abuse, physical, and emotional abuse on NSSI (*p* < 0.01), with physical abuse having the largest effect (14.82%, *p* < 0.001), followed by childhood abuse (9.65%, *p* < 0.001), and emotional abuse (8.12%, *p* < 0.001) in adjusted models. Social support had no mediating effect on the relationship between sexual abuse and NSSI. Among non-only children, there were no mediating effects between multiple forms of childhood abuse on NSSI in adjusted models.

When classifying sources of social support, controlling for confounding factors, all kinds of social support mediated the relationships between childhood abuse, physical abuse, emotional abuse, and NSSI in only-children, but none of the social support forms mediated the effect of abuse on NSSI in non-only children. Moreover, the mediating effect of social support from family and relatives is relatively larger than that from other sources. (See Appendix A, Table A1, Table A2, Table A3).

## 4. Discussion

Findings from this study show that 11.8% of the college students reported NSSI at least once (a higher rate of NSSI in only-children 13.4% than non-only children 11.1%), which is slightly lower than previous studies concerning Chinese adolescents and college students [4,30], but much higher than studies in the United States [31,32]. NSSI is a public health problem that cannot be ignored by college students, which seriously damages their physical and mental health. Studies have shown that early NSSI can lead to suicidal behavior and other mental illnesses later in life [33,34,35].

The current study found that physical, emotional, sexual, and overall childhood abuse scores were positively associated with NSSI frequency and negatively correlated with social support scores. Further, there was also a negative correlation between social support scores and the frequency of NSSI occurrence, which is fairly consistent with other studies. Previous research has demonstrated that abuse experiences during childhood are significantly associated with increased risk of NSSI in adolescents [10,26]. There was a negative correlation between childhood abuse and social support [36,37]. A prospective cohort design study showed that individuals with documented histories of child abuse reported significantly lower levels of social support in adulthood after adjusting for age, sex, and race [37]. Moreover, an investigation in a national representative sample of 4718 persons born in 1984 suggested that increasing social support may reduce the likelihood of NSSI in teenagers and young adults [18]. Moreover, it would appear that if the perpetrator of the abuse is a family member and the child is in a single child family, this may well influence the capacity to develop social support structures and influence the long-term outcome. One study found that childhood physical maltreatment and perceived social isolation in adulthood were associated with greater levels of internalizing symptoms in adulthood [38]. In addition, where childhood abuse exists in a family, intimate partner violence is also not uncommon, potentially further impacting on relationship formation and the challenges of developing autonomy, a process that requires parental support [39].

A substantial body of research has shown that childhood abuse can not only directly affect the occurrence of NSSI but also indirectly affect it through certain intermediary factors [40,41]. The results of this study indicate that social support does act as a mediator between childhood abuse and NSSI, especially among only-children. The college students who had experienced childhood abuse had an increased risk of NSSI, but the strength of this correlation decreased for those individuals that reported experiencing social support. However, the mediation effect of social support in the college student sample is smaller than that in middle school students [42]. One possible explanation may be that college students may be more independent compared to middle school students, so the intermediary effect of social support is reduced.

Our study further supported the claim that the mediating effect is mainly seen in the only-child population. Although, after adjusting for confounding factors, the mediating effect was significant and increased to 8.12%–14.82%, except for that between sexual abuse and NSSI. This is similar to Paivio SC’s study, in that sexual abuse considered alone was not significantly associated with the alexithymia, which precluded testing for mediational effects [43]. Therefore, the role of social support between sexual abuse and NSSI needs further study. However, strengthening social support did not significantly reduce the incidence of NSSI in non-only children. It may be related to the characteristics of only-children in that they show a more self-centered personality and a lower ability to integrate into the social group [42].

According to the results of different sources of social support in our study, the social support from parents and relatives had a greater mediating effect, compared to other sources of social support. This is consistent with previous results that only-children receive more attention from their parents, such as time, energy, and family assets, while the support from friends, classmates, and others is less [44,45,46,47]. As previously discussed, researchers have found evidence that individuals’ cognition of social support is negatively correlated with children’s emotional behavior problems. That is, the higher the level of social support children can perceive, the less emotional behavior problems they will have [48]. In other words, childhood abuse may change an individual’s ability to perceive the level of social support, and thus affect the behavior of NSSI. Therefore, the mediating effect of social support may be related to the individual’s cognition of social support, in that the higher level of social support perceived by the individual, the lower the possibility of NSSI. However, whether there are differences in individuals’ perception of social support also remains to be further studied among populations of only-children and among medical college students.

These findings indicated that the role of social support, especially that from parents and relatives, should be noted in only-children college students who have experienced childhood abuse. Moreover, educational settings are likely to represent an important conduit through which to improve the quality and accessibility of social support available to college students to enhance the perceptions and utilization of student social support and strengthen the awareness of school-community-families on children's adverse experiences and long-term adverse health effects. In view of this, intervention and prevention strategies focused on enhancing perceived social support as a fundamental feature, particularly among only child college students with a history of childhood abuse, may go some way toward mitigating the negative trajectory of childhood abuse in this population.

### Strengths and Limitations of the Study

Findings from this study underscore the importance of addressing social support underlying NSSI when developing NSSI interventions for young adults who have been maltreated. To the best of our knowledge, this study is the first to examine whether the only child status moderates the mediating effects of social support between childhood abuse and NSSI in Chinese undergraduate students. However, the study has some limitations. First, the study was cross-sectional; therefore, it is difficult to establish a causal relationship. Future studies should be more longitudinal in nature, which will provide a stronger understanding of causality; identifying and analyzing available longitudinal data with measures of childhood abuse outcomes, social support, and NSSI would provide a good opportunity to learn more about causal sequences among these variables. Second, it is possible that recall bias may exist with using self-reported questionnaires for data collection purposes. Third, some items of adverse childhood experience and NSSI are sensitive, which may affect the authenticity of participants’ information.

## 5. Conclusions

In conclusion, this study suggests that social support plays a mediating role in the association between childhood abuse and NSSI among only-children. As children grow up, parents, teachers, and classmates, as important components of the social environment, should give them more support and care, which may be beneficial to the prevention and control of NSSI.

## Figures and Tables

**Table 1:** Characteristics of participants by only-child, data shown as *n* (%)/*M*(*SD*).

Variable	Total (*n* = 4799)	Only Child (*n* = 1577)	Non-Only Child (*n* = 3222)	*p*-Value
Age (mean, s.d)	20.51 (1.02)	20.24 (0.87)	20.64 (1.06)	*p* < 0.001
Gender (*n* (%))				
boy	2011 (41.9)	903 (57.3)	1108 (34.4)	*p* < 0.001
girl	2788 (58.1)	674 (42.7)	2114 (65.6)	
Urban/rural				
rural	3139 (65.4)	532 (33.7)	2607 (80.9)	*p* < 0.001
urban	1660 (34.6)	1045 (66.3)	615 (19.1)	
Father’s education level				
Less than junior middle school	936 (19.5)	229 (14.5)	707 (21.9)	*p* < 0.001
Junior middle school	2184 (45.5)	464 (29.4)	1720 (53.4)	
Senior middle school	1051 (21.9)	412 (26.1)	639 (19.8)	
College or more	628 (13.1)	472 (29.9)	156 (4.8)	
Mother’s education level				
Less than junior middle school	2179 (45.4)	362 (23.0)	1817 (56.4)	*p* < 0.001
Junior middle school	1630 (34.0)	530 (33.6)	1100 (34.1)	
Senior middle school	655 (13.6)	414 (26.2)	241 (7.5)	
College or more	335 (7.0)	271 (17.2)	64 (2.0)	
Economic status of family				
poor	1339 (27.9)	240 (15.2)	1099 (34.1)	*p* < 0.001
moderate	3181 (66.3)	1169 (74.1)	2012 (62.4)	
good	279 (5.8)	168 (10.7)	111 (3.4)	
Non-suicidal self-injury ^#^				
Yes	568 (11.8)	211 (13.4)	357 (11.1)	0.021
No	4231 (88.2)	1366 (86.6)	2865 (88.9)	
Psychological symptoms (mean, s.d)	3.69 (6.61)	3.963 (7.10)	3.58 (6.35)	0.086
Childhood abuse (mean, s.d)				
Total	17.12 (5.26)	17.08 (5.02)	17.13 (5.37)	0.767
Physical abuse	7.21 (3.19)	7.25 (3.07)	7.19 (3.25)	0.524
Emotional abuse	5.64 (2.32)	5.53 (2.25)	5.69 (2.35)	0.024
Sexual abuse	4.27 (0.87)	4.30 (1.05)	4.25 (0.77)	0.060
Social support (mean, s.d)				
Total	32.10 (13.49)	30.86 (13.43)	32.70 (13.48)	*p* < 0.001
Classmates and friends	11.88 (5.34)	11.38 (5.31)	12.12 (5.34)	*p* < 0.001
Family and relatives	12.32 (5.57)	11.88 (5.64)	12.53 (5.52)	*p* < 0.001
Others	8.36 (4.01)	8.11 (3.95)	8.48 (4.03)	0.003

^#^ The frequency of NSSI more than 0.

**Table 2:** Correlation analysis of child abuse, social support, and NSSI.

Category	Variable	CA	PA	3. EA	4. SA	5. SS	6. NSSI
Total	1. Childhood abuse	1.00					
	2. Physical abuse	0.92 *	1.00				
	3. Emotional abuse	0.86 *	0.63 *	1.00			
	4. Sexual abuse	0.39 *	0.19 *	0.25 *	1.00		
	5. Social support	−0.20 *	−0.17 *	−0.20 *	−0.08 *	1.00	
	6. NSSI	0.27 *	0.22 *	0.28 *	0.11 *	−0.11 *	1.00
Only child	1. Childhood abuse	1.00					
	2. Physical abuse	0.90 *	1.00				
	3. Emotional abuse	0.83 *	0.59 *	1.00			
	4. Sexual abuse	0.36 *	0.14 *	0.14 *	1.00		
	5. Social support	−0.19 *	−0.17 *	−0.18 *	−0.003	1.00	
	6. NSSI	0.19 *	0.13 *	0.22 *	0.07 *	−0.16 *	1.00
Non-only child	1.Childhood abuse	1.00					
	2. Physical abuse	0.92 *	1.00				
	3. Emotional abuse	0.88 *	0.65 *	1.00			
	4. Sexual abuse	0.43 *	0.23 *	0.33 *	1.00		
	5. Social support	−0.21 *	−0.17 *	−0.21 *	−0.13 *	1.00	
	6. NSSI	0.31 *	0.26 *	0.32 *	0.13 *	−0.08 *	1.00

* The correlation was significant at the 0.01 level (bilateral); CA = childhood abuse; PA = physical abuse; EA = emotional abuse; SA = sexual abuse; SS = social support; NSSI = non-suicidal self-injury.

**Table 3:** Mediation effects of social support between CA and NSSI in the full sample.

Types of CA	Model	Boot *LLCI*	Boot *ULCI*	Indirect Effect a × b	Boot *LLCI*	Boot *ULCI*	Direct Effect c’	Mediation Ratio, % a × b/(a × b + c’)
Childhood abuse	1	0.0022	0.0097	0.0057	0.1254	0.1550	0.1402 ^**^	3.91%
	2	−0.0009	0.0037	0.0012	0.1088	0.1393	0.1241 ^**^	ND
Physical abuse	1	0.0055	0.0164	0.0104	0.1555	0.2047	0.1801 ^**^	5.46%
	2	−0.0003	0.0061	0.0025	0.1302	0.1800	0.1551 ^**^	ND
Emotional abuse	1	0.0054	0.0206	0.0124	0.2963	0.3634	0.3298 ^**^	3.62%
	2	−0.0028	0.0078	0.0023	0.2636	0.3318	0.2977 ^**^	ND
Sexual abuse	1	0.0115	0.0408	0.0237	0.2263	0.4066	0.3164 ^**^	6.97%
	2	−0.0046	0.0081	0.0010	0.1139	0.2960	0.2049 ^**^	ND

^**^p < 0.001; Model 1: Unadjusted analysis; Model 2: Adjusted for age, gender, registered residence, parents’ education, perceived family economic status, psychological symptoms.

**Table 4:** Mediation effects of social support between CA and NSSI in only-children.

Types of CA	Model	Boot *LLCI*	Boot *ULCI*	Indirect effect a × b	Boot *LLCI*	Boot *ULCI*	Direct Effect c’	Mediation Ratio, % a × b/(a × b + c’)
Childhood abuse	1	0.0067	0.0248	0.0138	0.0691	0.1257	0.0974 ^**^	12.41%
	2	0.0032	0.0152	0.0077	0.0431	0.1012	0.0721 ^**^	9.65%
Physical abuse	1	0.0113	0.0409	0.0226	0.0541	0.1471	0.1006 ^**^	18.34%
	2	0.0053	0.0251	0.0127	0.0262	0.1198	0.0730^*^	14.82%
Emotional abuse	1	0.0150	0.0512	0.0294	0.1890	0.3148	0.2519 ^**^	10.45%
	2	0.0075	0.0342	0.0178	0.1373	0.2653	0.2013 ^**^	8.12%
Sexual abuse	1	−0.0281	0.0373	0.0013	0.0648	0.3327	0.1988^*^	ND
	2	−0.0554	0.0009	−0.0218	−0.0784	0.1941	0.0578	ND

^**^p < 0.001; ^*^p < 0.01; Model 1: Unadjusted analysis; Model 2: Adjusted for age, gender, registered residence, parents’ education, perceived family economic status, psychological symptoms.

**Table 5:** Mediation effects of social support between CA and NSSI in non-only children.

Types of CA	Model	Boot *LLCI*	Boot *ULCI*	Indirect Effect a × b	Boot *LLCI*	Boot *ULCI*	Direct Effect c’	Mediation ratio, % a × b/(a × b + c’)
Childhood abuse	1	−0.0021	0.0056	0.0016	0.1420	0.1764	0.1592 ^**^	ND
	2	−0.0043	−0.0011	−0.0013	0.1276	0.1630	0.1453 ^**^	ND
Physical abuse	1	0.0007	0.0110	0.0054	0.1858	0.2432	0.2145 ^**^	2.46%
	2	−0.0043	0.0024	−0.0006	0.1628	0.2211	0.1920 ^**^	ND
Emotional abuse	1	−0.0046	0.0120	0.0036	0.3281	0.4068	0.3674 ^**^	ND
	2	−0.0105	0.0020	−0.0036	0.2974	0.3775	0.3374 ^**^	ND
Sexual abuse	1	0.0132	0.0535	0.0295	0.3133	0.5601	0.4367 ^**^	6.33%
	2	−0.0090	0.0123	0.0011	0.2032	0.4514	0.3273 ^**^	ND

^**^p < 0.001; Model 1: Unadjusted analysis; Model 2: Adjusted for age, gender, registered residence, parents’ education, perceived family economic status, and psychological symptoms.

## Appendix A

**Table A1:** Childhood abuse - social support from classmates and friends - NSSI.

Types of CA	Only Child	Boot *LLCI*	Boot *ULCI*	Indirect Effect a × b	Boot *LLCI*	Boot *ULCI*	Direct Effect c’	Mediation Ratio, % a × b/(a × b + c’)
Childhood abuse	Yes	0.0019	0.0113	0.0053	0.0456	0.1035	0.0745 ^**^	6.64%
	No	−0.0023	0.0030	0.0003	0.1260	0.1614	0.1437 ^**^	ND
Physical abuse	Yes	0.0029	0.0175	0.0081	0.0310	0.1242	0.0776^*^	9.45%
	No	−0.0014	0.0049	0.0014	0.1608	0.2190	0.1899 ^**^	ND
Emotional abuse	Yes	0.0061	0.0301	0.0152	0.1400	0.2679	0.2039 ^**^	6.94%
	No	−0.0066	0.0060	−0.0002	0.2939	0.3743	0.3341 ^**^	ND
Sexual abuse	Yes	−0.0612	−0.0100	−0.0290	−0.0717	0.2016	0.0650	ND
	No	−0.0022	0.0248	0.0084	0.1958	0.4441	0.3200 ^**^	ND

^**^*p* < 0.001; ^*^*p* < 0.01. Adjusted for age, gender, registered residence, parents’ education, perceived family economic status, and psychological symptoms.

**Table A2:** Childhood abuse - social support from family and relatives - NSSI.

Types of CA	Only Child	Boot *LLCI*	Boot *ULCI*	Indirect Effect a × b	Boot *LLCI*	Boot *ULCI*	Direct Effect c’	Mediation Ratio, % a × b/(a × b + c’)
Childhood abuse	Yes	0.0035	0.0176	0.0086	0.0421	0.1004	0.0713 ^**^	10.78%
	No	−0.0057	−0.0001	−0.0024	0.1287	0.1641	0.1464 ^**^	ND
Physical abuse	Yes	0.0059	0.0286	0.0142	0.0247	0.1185	0.0716^*^	16.57%
	No	−0.0064	−0.0006	−0.0021	0.1643	0.2225	0.1934 ^**^	ND
Emotional abuse	Yes	0.0068	0.0369	0.0174	0.1377	0.2656	0.2017 ^**^	7.94%
	No	−0.0136	−0.0008	−0.0058	0.2997	0.3797	0.3397 ^**^	ND
Sexual abuse	Yes	−0.0365	0.0192	−0.0077	−0.0922	0.1797	0.0437	ND
	No	−0.0173	0.0052	−0.0036	0.2078	0.4561	0.3319 ^**^	ND

^**^*p* < 0.001; ^*^*p* < 0.01. Adjusted for age, gender, registered residence, parents’ education, perceived family economic status, and psychological symptoms.

**Table A3:** Childhood abuse - social support from others - NSSI.

Types of CA	Only Child	Boot *LLCI*	Boot *ULCI*	Indirect Effect a × b	Boot *LLCI*	Boot *ULCI*	Direct Effect c’	Mediation Ratio, % a × b/(a × b + c’)
Childhood abuse	Yes	0.0018	0.0134	0.0061	0.0447	0.1028	0.0737 ^**^	7.64%
	No	−0.0014	0.0016	0.0000	0.1264	0.1616	0.1440 ^**^	ND
Physical abuse	Yes	0.0034	0.0243	0.0109	0.0279	0.1217	0.0748^*^	12.72%
	No	−0.0016	0.0029	−0.0003	0.1620	0.2201	0.1910 ^**^	ND
Emotional abuse	Yes	0.0042	0.0281	0.0132	0.1420	0.2699	0.2059 ^**^	6.02%
	No	−0.0038	0.0036	−0.0002	0.2942	0.3740	0.3341 ^**^	ND
Sexual abuse	Yes	−0.0539	−0.0036	−0.0211	−0.0795	0.1937	0.0571	ND
	No	−0.0050	0.0029	0.0001	0.2047	0.4523	0.3285 ^**^	ND

^**^*p* < 0.001; ^*^*p* < 0.01. Adjusted for age, gender, registered residence, parents’ education, perceived family economic status, and psychological symptoms.
